# Problem-based learning in research method courses: development, application and evaluation

**DOI:** 10.12688/f1000research.75985.2

**Published:** 2023-12-11

**Authors:** Yani Ramdani, Nia Kurniati Syam, Yayat Karyana, Diar Herawati

**Affiliations:** 1 Mathematics Study Program of The Faculty of Mathematics And Natural Sciences, Universitas Islam Bandung, Bandung, 40116, Indonesia; 2 Islamic Communication and Broadcasting Faculty of Da'wah, Universitas Islam Bandung, Bandung, 40116, Indonesia; 3 Statistics Study Program of The Faculty of Mathematics And Natural Sciences, Universitas Islam Bandung, Bandung, 40116, Indonesia; 4 Pharmacy Study Program of Faculty of Mathematics and Natural Sciences, Universitas Islam Bandung, Bandung, 40116, Indonesia

**Keywords:** problem-based learning, courses of research methods, learning outcomes, scientific reports

## Abstract

**Background:**

The undergraduate curriculum in Indonesia generally requires students to take a research methods course as a prerequisite course for the preparation of scientific reports. The objective of this course is to teach students how to present research results both orally and in writing using the styles and forms of each university. However, this course is not popular among students because the material is complex and technical. As a result, there is a gap between learning outcomes and their application when students prepare scientific reports. Improving pedagogy and teaching interventions through student-developed research projects is important in complementing lectures. The purpose of this study was to analyze the improvement of students’ ability in writing scientific reports through research method courses using problem based learning (PBL).

**Methods:**

This research was a case study to report the integration of scientific report writing in research methods courses through PBL at the Universitas Islam Bandung in Indonesia. PBL was implemented by involving students in research-related tasks with the following stages: writing research questions, determining the research design, collecting data, analyzing data, and presenting research results.

**Results:**

The research results showed that there was an increase in scientific writing skills in the good category. Normalized gain for indicators of increasing accuracy using research report formats, suitability for using literature and research methods is 0.064 and 0.209, including the low category, and the plagiarism level is 0.509, including the medium category.

**Conclusions:**

This finding implies that assigning students to write scientific reports using PBL in the research method classroom is useful for improving learning outcomes, presentation, reasoning, communication, ambiguous problem-solving, and reducing plagiarism. The findings of this study strengthen the findings of previous researchers, namely increase students’ abilities in using formatting to prepare scientific reports, using appropriate literature, mastering research materials as well as reducing the level of plagiarism.

## Introduction

Universities have an important role in generating new knowledge through scientific writing to develop science and create innovation in order to improve people’s lives. Scientific writing is an essential competency for a career in science. Many scientific writing techniques are contained in research methods courses. However, this course is usually less attractive to students because the material is complex and technical. ^1^Students often do not understand the purpose of this course, so they follow it with trepidation and skepticism. ^2^^,^^3^ As a result, students still find it difficult to apply what they have learned in research methods lectures in the preparation of proposals and thesis reports. ^4^ Some scholars state that conventional lecture-based teaching is not effective in overcoming barriers to understanding and applying research methods. ^5^ Improving pedagogy and teaching interventions in research methods courses is important. ^6^^–^^8^ There are suggestions for implementing learning to develop competencies that allow students to make strategies to find new knowledge and the ability to solve problems. ^9^ Some of the suggested learning techniques are problem-based learning (PBL), ^10^ exploratory data analysis, ^11^ and learning focused on research articles. ^12^^,^^13^

The Indonesian government regulation through research and higher education technology ( *Peraturan Menteri Riset Teknologi dan Pendidikan Tinggi/MENRISTEKDIKTI)* number 44, year 2015 states that to be able to realize graduate competency standards, the lecture model developed must be interactive, holistic, integrative, scientific, contextual, thematic, effective, collaborative, and student-centered. ^14^ One of the learning models that meet these characteristics is problem-based learning (PBL). PBL is a learning model to develop competence. ^15^ It is collaborative and student-centered and requires students to play an active role in re-discovering knowledge by applying the principles of constructivism based on their initial knowledge. ^12^ This learning model promotes the exploration of new knowledge and is integrated with different courses. ^15^

This paper explores the results of basic research on the application of the PBL method in a research methods course in a mathematics study program at an Universitas Islam Bandung in Indonesia. In general, in the curriculum for the Bachelor Program (S1) in Indonesia, every student must write a scientific paper called a thesis to complete their study period. To equip students in thesis writing, students must take a research methods course. The general objective of this course is to teach students how to present research results both orally and in writing using the style and form of each university. PBL allows students to construct new knowledge by relating their prior knowledge more easily. ^16^ PBL allows students to search for information, solve problems, make decisions, work in groups, write reports, make presentations, be independent and responsible in dealing with complex problems from real life. ^17^

PBL is thought to be the right learning method to create an awareness of the important role of understanding and applying research methods for thesis completion and career development. The objective of this research was to analyze the application of the PBL method in improving the competence of students in preparing scientific reports. The indicators measured were the level of plagiarism using Turnitin, use of scientific report preparation formats, literature suitability and mastery of research report content in in-class presentations.

### Higher education problem-based learning

The characteristics of the learning process in Permenristekdikti no 44 of 2015 article 11 consist of the following characteristics:

- Interactive – the learning outcomes of graduates are achieved by prioritizing the two-way interaction process between students and lecturers through offline and online teaching and learning processes.
- Holistic – the learning process encourages the formation of a comprehensive mindset by internalizing local and national excellence and wisdom.
- Integrative – the learning outcomes of graduates are achieved through an integrated learning process to meet the overall learning outcomes of graduates in one multidisciplinary process.
- Scientific – graduate learning outcomes are achieved through a learning process that prioritizes a scientific approach to create an academic environment that is based on a system of values, norms, and scientific principles and upholds religious and national values.
- Contextual – graduate learning outcomes are achieved through a learning process that is adapted to the demands of the ability to solve problems in the realm of expertise.
- Thematic – graduate learning outcomes are achieved through a learning process that is adapted to the scientific characteristics of the study program and is linked to real problems through a transdisciplinary approach that is implemented through structured assignments where students are trained to solve problems taken from everyday life.
- Effective – the learning outcomes of graduates are achieved effectively by emphasizing the internalization of the material properly and correctly in an optimum period.
- Collaborative – graduate learning outcomes are achieved through a shared learning process that involves interaction between individual learners to produce the capitalization of attitudes, knowledge, and skills that are implemented on student projects as a group.
- Student-centered – graduate learning outcomes are achieved through a learning process that prioritizes the development of creativity, capacity, personality, and student needs, as well as developing independence in seeking and finding the knowledge by applying student-centered learning methods such as problem-based learning, contextual teaching, and learning.

The above characteristics are in line with the PBL method. PBL places more emphasis on active, interactive, and collaborative learning, problem resolution, and decision-making providing opportunities for independent study and presentation ^18^^,^^19^ to develop critical thinking and analytical skills ^20^^,^^21^ The PBL method has advantages over conventional learning. ^22^ This learning model has been widely applied in universities and is used to develop skills needed by the jobs market such as group work and relationships, as well as collaborative, proactive and entrepreneurial skills ^23^^,^^24^ in the field of engineering, ^25^^–^^28^ medicine, ^29^^,^^30^ economics, ^31^ pharmaceuticals, ^32^ psychology ^33^ and others.

The main objectives of the PBL course are (1) to encourage independent learning in students, which leads to higher motivation, better retention of material, development of reasoning, and problem-solving abilities, and (2) to develop a better understanding in students of the process and the skills necessary for successful work collaboration. ^1^ There are similarities between PBL goals and research methods learning objectives that we have in undergraduate mathematics programs. The field of applied mathematics can be more interesting by using PBL as an alternative methodology to deal with current and future problems. ^24^ The learning process in mathematics generally requires good reasoning skills. The PBL approach has been developed to improve students’ reasoning abilities. ^30^

The purpose of implementing PBL in the research method classroom was to measure students’ ability to write scientific reports through problems that must be solved so as to encourage students to learn actively, have independent learning, and be able to apply mathematical problems in mathematics, mathematics in other fields, and mathematics in real life.

## Methods

### Ethics approval

This study was approved by Universitas Islam Bandung (Nomor: 500/B.04/Bak-k/XII/2019) after due consultation and all participants provided their written informed consent.

### Participants

The participants were third-year students at an Universitas Islam Bandung Indonesia (a total of 40 students including nine males and thirty-one females, thirteen of whom had high abilities, eighteen were moderately capable and nine had low abilities. Information on the grouping based on gender and students’ ability levels was obtained from the Cumulative Achievement Index/GPA) enrolled in the courses of research methods. The details are presented in Table 1 and Table 2.

**Table 1.** Data description based gender.

Gender	Frequency	Relative frequency (%)
Male	9	22.5
Female	31	77.5
Total	40	100

**Table 2.** Data description based ability.

Ability	Frequency	Relative frequency (%)
High	13	32.5
Middle	18	45.0
Low	9	22.5
Total	40	100

In general, each student had different abilities in thinking and communicating. One of the differences could be seen from the value of the GPA. GPA was the measure performance of student at the academic field which was obtained by combining all the grades of the courses that had been taken up to a certain semester. ^34^ All students met the requirements agreed to: (1) case presentation, (2) conduct discussions, (3) make a summary, (4) participate in online learning, and (5) compile scientific reports.

### PBL teaching process

Students and researchers in the field of science are generally trained and motivated to design, conduct experiments, and analyze data. ^35^ PBL was carried out in three stages, namely preparation, implementation, and evaluation. First, a semester lesson plan with learning sub-achievements was developed so students could understand the meaning of research, definitions, and methods, explain research and decision-making processes, make systematic decisions, formulate research problems, present a literature review, develop a theoretical framework and formulate hypotheses, design a study and compile research proposals. Integration of research proposal writing in research methods courses was claimed to play a role in improving student research learning, ^36^ selecting representative research samples, collecting data appropriately, measuring and designing surveys, conducting data analysis and descriptive studies and compiling reports study. Second, the case determination scenario was based on the areas of specialization determined by the mathematics study program, namely: (1) financial and industrial mathematics; (2) mathematics computer science. Research topics were then developed based on research titles proposed by students to encourage class discussion. Arguably, the most popular active learning experience in research methods courses is student-developed research projects. ^37^ The implementation of PBL was carried out in three stages, namely case presentations, discussions, and compiling research reports. Third, evaluation: the preparation of research reports was done individually, the similarity test was determined not to be more than 25%. Students received a very good score (A) if they successfully used the report preparation format correctly, used appropriate literature, there was a maximum plagiarism rate of 25% and they mastered the content of research reports through presentations in class. On the other hand, students received a very poor score (D and E) if they reviewed research reports without showing proper understanding.

### PBL design and preparation

This course was designed for students who would write a final year project in mathematics. The material provided is an introduction to mathematical theory and applied mathematics, research paradigms in mathematics and their applications, and how the ontological and epistemological assumptions used by researchers affect research methods. ^31^ This course teaches an introduction to research design and relevant research methods in the field of mathematics. It includes a discussion of various methods that can be used to solve mathematical problems and their application. Each student conducted small-scale research which was used as a pilot in the final undergraduate project. The course must be taken in the previous semester or in the same semester when students work on their final project. Lectures are held for 100 minutes every week for 16 weeks in one semester. Students were divided into eight groups. The grouping of students was based on research topics proposed by students, namely: pure mathematics (analysis and algebra) and applied mathematics (economics, industry, and computers). Each student was allocated 20 minutes for the presentation. The role of the lecturer was the facilitator during the discussion.

PBL activity

Research method learning was held in the form of lectures and seminars. Teaching materials were mostly related to their small-scale research assignments. One week before the implementation of PBL, the lecturer assigned students to do homework according to research topics such as (1) searching and reading information on websites, reading the results of scientific research focusing on the goals and objectives of students’ specific research, for example, articles in published journals and books. (2) collecting 14 relevant articles and (3) making a table of other peoples’ research results containing references, abstracts, conclusions, and suggestions. The task table was used to inspire the background, objectives, methodology, findings, significance, follow-up, and references of the research. Such tasks play an important role in developing students’ learning and interest in conducting research. ^38^^,^^39^ This research activity applies the five research phases namely: (1) writing research questions, (2) determining the research design, (3) collecting data, (4) analyzing data, and (5) presentation of research results. In general, the five-phase process for this student research project allows for the allocation of time for each phase, on average one week, except for the research methodology. ^1^

Phase 1: Creation of research questions. Students were assigned to review 14 articles that were relevant in terms of theory and method and then make a summary in tabular form. This assignment was expected to generate new ideas so that research topics could be obtained. Lecturers grouped the topics of articles collected by students into groups of specialization fields, namely, pure mathematics (analysis and algebra) and applied mathematics (economics, industry, and computers). Students formed groups based on the area of interest and brainstormed some research questions to be investigated individually. Lecturers acted as facilitators of brainstorming activities through class discussions. Each group wrote each research question proposed by each student on the blackboard. Students reviewed their ideas outside of class. This activity was carried out to avoid overlapping questions.

Phase 2: Research design. The students formulated the problem and research objectives and they were approved by the lecturer. The lecturer then equipped students with materials for developing theoretical frameworks, formulating hypotheses, planning research, and compiling research proposals. Students compiled a literature review as well as a bibliography on their research proposals by examining theories that strengthen the research methods used.

Phase 3: Data collection. Lectures were given on techniques for selecting representative research samples, collecting data correctly, and techniques for measuring and designing surveys. Data could be obtained by students through the Internet, books, articles, or theses. Students used these data by, for example, comparing two methods. This activity aimed to prevent students from plagiarizing other work.

Phase 4: Data processing and analysis. Students prepared data files, processed data according to settlement methods, conducted consultations, discussed the results of data processing and analyses and interpreted the findings. The limited experience of students doing data analysis encouraged lecturers to play an important role providing confidence about the accuracy of student findings.

Phase 5: Presentation of research results. The lecturer provided direction for the entire class to prepare presentation materials including publication style, article format, PowerPoint techniques, speaking style, presentation skills, and optimization of presentation time. Each student was given 20 minutes of presentation time followed by 10 minutes of discussion. Lecturers acted as facilitators who directed and validated findings. Research questions posed by students are presented in Table 3.

**Table 3.** The research questions proposed by students.

1	Searching for an effective route for shipping goods by looking for a minimum spanning tree using the Kruskal Algorithm. The case study discussed in the search for shipping routes from SiCepat Express Baleendah to 15 sub-districts in Bandung Regency. The 15 sub-districts referred to include Baleendah, Dayeuhkolot, Bojongsoang, Banjaran, Ciparay, Arjasari, Margahayu, Katapang, Majalaya, Pameungpeuk, Soreang, Margaasih, Cileunyi, Solokanjeruk and Rancaekek sub-districts. The final result that must be obtained is in the form of a connected graph whose minimum spanning tree has been determined, the route of delivery of goods, and the shortest distance from SiCepat Express Baleendah to the 15 sub-districts.
2	Maple implementation in solving problems of integral application and its comparison with manual work.
3	Analysis of damped spring vibration model using Runge-Kutta gill. method.
4	Application of the Milne method to analyze the solution to the damped spring vibration model.
5	Application of differential equations in modeling population growth in the city of Bandung.
6	The effect of online shopping budget planning on the amount of finance each month for students at the Islamic University of Bandung.
7	The formation of an optimal portfolio using the single index model method in the property, real estate, and building construction sector of the city of Bandung.
8	Optimization of the 2021 MotoGP schedule with a modified algorithm from the Prim algorithm in terms of the distance between circuits.
9	Optimal portfolio modeling using Single Index Model on LQ-45 index companies on the Indonesia Stock Exchange.
10	Comparative analysis of the method of calculating interest on installment sales for Honda Scoopy sporty motorbikes on Honda Cengkareng.
11	Analysis of risk management methods in making investment decisions during the pandemic (a case study of PT FAPA AGRI Tbk).
12	The influence of covid-19 on the Grade Point Average (GPA) of Unisba mathematics students.
13	A study of the mathematical perspective of Islamic insurance in Islam.
14	Optimal portfolio analysis of single index model for property and real estate companies in Bandung.
15	Modeling the influence of income and number of family members on household consumption patterns using multiple linear analysis models with case studies in two sub-districts, namely the Darangdan sub-district and Bojong sub-district.
16	Optimizing food products at the Uni Dona Padang restaurant using the Simplex Method.
17	The use of GeoGebra software media for linear programming learning in optimizing the profits of mango sticky rice production (a case study of Chachamango Bandung).
18	Comparison of the risk and level of risk of LQ 45 and Non-LQ 45 shares in companies listed on the Indonesian Stock Exchange (IDX) using the single index method.
19	The simplex method in optimizing the production of poor baso meatballs in the Lembang area of Bandung.
20	The effect of return on assets (ROA) and return on equity (ROE) on profitability and financial performance at PT Bank Central Asia Tbk.
21	Financial performance at PT. Pos Indonesia in terms of financial ratio analysis using the Liquidity Ratio analysis method.
22	The use of a single index method in the formation of an optimal portfolio (study on banking stocks listed in IDX30 for the period February 2018 – August 2020).
23	Optimization of the shortest path using the Kruskal Algorithm for garbage collection at Mekarsari Permai housing, Bandung.
24	Implementation of modulo arithmetic for finding notes in a chord.
25	Application of the Simple Additive Weighting (SAW) method in determining customers who are eligible for microfinance based on the value of the collateral.
26	Raw material inventory control using the EOQ method at UD Adi Mabel.
27	The insurance calculation based on the rainfall index uses the burn analysis method to determine a fair premium value.
28	Forecasting fish production using the POM-QM application for Windows with the Linear Trend Line Model method in West Java.
29	Determination of stock composition using a single index method informing the optimal portfolio of stocks to determine the optimal portfolio candidate in the 2010-2014 period.
30	The application of a decision support system using the Topsis method to determine the increase in the class of students at the Al-Falah Islamic Boarding School Dago Bandung.
31	Analysis of loan interest rates based on installment payments.
32	Mathematical communication, problem-solving and critical thinking to reduce anxiety about learning mathematics.
33	Mathematical modeling using the application of ordinary differential equations to predict the rate of population growth in Indonesia.
34	Application of value at risk management in online business.
35	Determining the best alternative types of fabric raw materials based on many alternatives and many criteria that exist in XYZ Convection Leggings with the VIKOR method to optimize convection profits without compromising customer satisfaction.
36	Build a minimum spanning tree to optimize the Bandung area's national transmission network by applying the Prim. Algorithm.
37	Analysis of the dynamics of HIV with a deterministic model using MATLAB.
38	Export Quality Control of Manggis Fruit Using Statistical Quality Control (SQC) (Case Study of PT. AB Exporter).
39	Analysis of Dammed Spring Vibration Model in Motorized Vehicles Using the Runge-Kutta Gill and Milne Method.
40	Implementation of Mamdani Fuzzy Inference in Determining the Recipient of Direct Cash Assistance in East Belitung District.

In the implementation of PBL, lecturers presented to the students the research topics they had proposed. Lecturers guide students to form groups based on keywords. The students in each group discussed and exchanged ideas and discussed theoretically the problems that would be selected in the research.

### Data collection and analysis

This study examined whether PBL can improve students’ competence in writing scientific reports with competencies developed including the level of plagiarism, accuracy of the use of research formats, suitability of the literature, and mastery of research material through presentations. The research hypotheses were as follows:

**Table:** 

H _1_:	There is an increase in the accuracy of using the format for preparing scientific research reports in the research method class with the application of PBL.
H _2_:	There is an increase in the suitability of using literature and the method of preparing scientific research reports in the research method class with the application of PBL.
H _3_:	There is a decrease in the level of plagiarism in the preparation of scientific reports in the research method class with the application of the PBL method.
H _4_:	There is an increase in the ability to present scientific research report material in the research method class with the application of PBL.
H _5_:	There is an increase in the competence of preparing scientific research reports in the research method class with the application of PBL.

The research data was taken from the results of the evaluation of student research reports with similarity test indicators using Turnitin, the use of the report preparation format, the suitability of the literature, mastery of the content of research reports in-class presentations. The research was conducted twice, namely in the mid-semester exam (week eight was evaluation of stage 1) and the final exam (week sixteen was evaluation of stage 2). The results of the mid-semester exam and the final exam can be found in the underlying data. ^40^ To increase students’ ability in compiling research reports, the normalized gain is used with the formula:

$$\text{Normalized gain}\;\left(g\right)=\frac{\text{final exam scores}-\text{midterm test scores}}{\text{ideal value}-\text{midterm test scores}}$$

With normalized gain categories (g) are: g < 0.3 is low; 0.3 g < 0.7 is moderate; and 0.7 g is high. Hypothesis testing used Independent sample *t*-test (df = 95) and Wilcoxon Signed Ranks Test, which compared the average score of each stage for each indicator compared with a significance value of α = 0.05. We used IBM SPSS statistics version 22 (RRID:SCR_019096).

## Results and discussion

**Figure 1.**
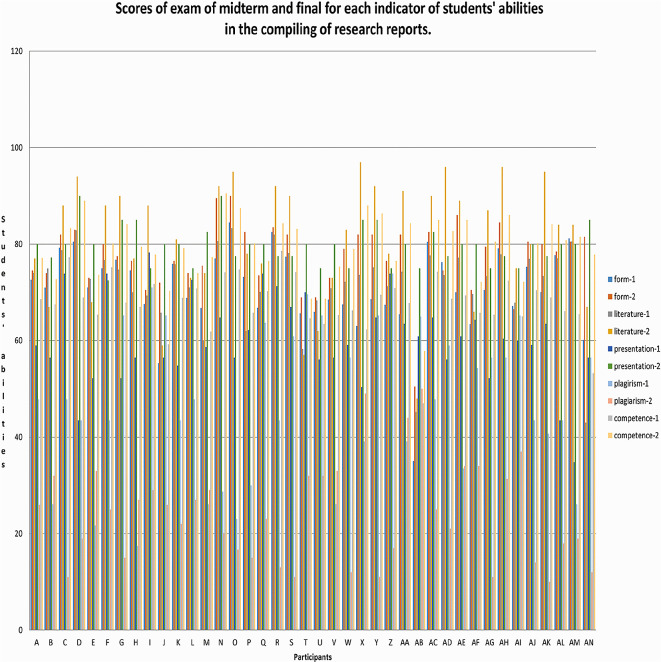
Mid-term and final exam scores for each indicator of students' abilities in the compiling of research reports.

This study involved 40 college students (nine boys and thirty-one girls) with an average age of twenty-one years. The age and gender of students did not have a significant effect on the competence of preparing research reports. The student’s ability level (high, medium, and low) was only associated with the cumulative achievement index and was not examined in this study because every student in the research method class must be able to complete the scientific report. The increase in accuracy using the research report format was *g* = 0.064 including the low category, suitability using literature and research methods was *g* = 0.209 including the low category, and plagiarism levels was *g* = 0.509 including the medium category. An overview of the research data is shown in Figure 1. Table 4 describes students’ abilities in compiling research reports.

**Table 4.** Descriptive statistics of students' abilities in the compiling of research reports.

Measurement indicator	N	Stage	Minimum	Maximum	Mean	Std. Deviation
The accuracy of using the research report format	40	Stage 1	35.00	84.50	70.8725	8.71451
		Stage 2	50.50	90.00	77.4500	7.01902
Suitability of using literature and research methods	40	Stage 1	43.00	83.30	72.0078	8.60646
		Stage 2	48.00	97.00	80.9250	12.22311
Possible plagiarism	40	Stage 1	34.80	78.30	60.3912	9.12268
		Stage 2	72.50	90.00	79.6187	4.02874
Mastery of the content of research reports in-class presentations	40	Stage 1	47.02	78.60	67.7572	5.92759
		Stage 2	57.83	90.50	79.3312	6.58157
Competency in preparing research reports	40	Stage 1	17.40	73.90	47.4800	15.76912
		Stage 2	10.00	50.00	24.1500	10.51943
Valid N (listwise)	40					

The results for stage one and stage two show that the accuracy of using the research report format, the suitability of using literature and research methods, presentation skills, and the competence of preparing research reports were normally distributed and homogeneous. While the level of similarity (plagiarism) stage one was normally distributed and stage two was not normally distributed. The results show that all hypotheses were rejected, meaning that there was a significant increase in the average score between stage one and stage two on indicators of accuracy using the research report format, suitability of using literature and research methods, presentation skills, and competence in preparing research reports with an average score at stage-1 higher than stage-2 and they fall in the good and very good categories. There is a significant decrease between the likelihood of plagiarism in stage one and stage two.

The results of the study indicated that the application of PBL in research methods courses could improve learning outcomes related to the preparation of research reports in good categories with indicators of possible plagiarism, use of report preparation formats, suitability of literature, and presentations. PBL resulted in significant improvements in learning outcomes, students’ perceptions of university social responsibility, their capacity to deal with complex and ambiguous structural problems, their ability to put professional knowledge into practice, team building, and communication skills. ^35^ The possibility of plagiarism was reduced because the research process was monitored during the creation of research topics, research proposals and the submission of research reports. ^3^ The PBL approach provides significant benefits for students in presentation skills. ^41^ PBL enables students to develop information seeking, problem-solving, decision making, group work skills. and other skills such as writing reports, making presentations, independent learning, being able to face and solve complex problems in real life. ^17^ PBL could improve learning achievement, problem solving skills, and interaction skills of students. ^42^ PBL experiments make it easy to link previous understanding of the material with new knowledge to increase the ability to construct knowledge for students.

This article discusses a simple way to overcome one of the difficulties of students in compiling research reports. Assigning the preparation of small-scale research reports through the PBL approach in the research methods class helps students apply the theory of research methods in solving the problems they face. This approach does not require special technology so that it can be used in various conditions without having to add commitment from the lecturer. However, it requires a higher level of student participation to contribute to discussions and other activities. This learning inspires students on research that is relevant for research in the field of mathematics and its applications. Students are trained to develop different research designs and methods in collecting, processing, and analyzing data. The goal is to enable each student to find ideas that lead to the emergence of researchable questions and to determine the appropriate method to answer research questions. Students are also encouraged to develop current research issues that can be linked to other courses to enable students to conduct scientific research for thesis writing. Research methods courses should be in the same semester as the preparation of the thesis so that the material can be directly implemented. In general, before the preparation of the thesis, each student already has a proposal supervisor so that the feasibility level of research assignments in learning research methods gets an assessment from the student proposal supervisor. Of course, empirical research that examines the quality of research products provided by students in the method course will help support this claim. Finally, further research is needed to test the product of their completed research. ^18^ Some criticisms of PBL raise concerns regarding the ability of lecturers to monitor and evaluate each student’s research project at the same time. The support needs in research writing are supervisory support, peer support, ^43^ skills, and research development support. ^44^ Supervision is required in three phases including 1) purification and completion of research proposals; 2) data creation; and 3) analyzing data and ‘writing it down’. ^45^ Peer support can take the form of a peer tutor. In addition, scientific writing support is recognized as an area that all students need. ^46^ Some PBL authors recommend the use of peer tutors to cope with demands on lecturers for larger classes. ^47^

This study is a single study so the findings may be equivocal. In the absence of a control group, the advantages of implementing PBL cannot be compared. In addition, a standardized evaluation system is needed to review the optimization of PBL implementation. However, the research results can be used as a basis for experimentation for future research with PBL applications because it has significant challenges to develop student competencies such as reasoning, communication, problem solving, learning community, preparation of research reports, and presentations. These skills are needed by future students.

## Conclusions

The results of this study indicate that the integration of research report writing through the PBL approach in the research method class can develop students’ abilities in compiling research reports with a significance value of 0.00. This integration is one of the best ways to engage and apply research methods theory directly in student research. The improvement in overall learning outcomes at the end of the positive PBL process indicates that integrated teaching is an effective way to reduce student barriers in compiling research reports. Students also realize that writing scientific reports is a learning process whose mastery of skills must be done through experience. Finally, some students feel trained and motivated in designing, conducting experiments, processing, and analyzing data. Based on the findings of this study, the PBL approach is recommended to support research at the undergraduate level. To ensure the quality of scientific reports, the support for scientific writing needs to get better attention. Supervision can involve other lecturers to form peer teaching. Peer support is developed to form a mutually supportive learning community.

## Data availability

### Underlying data

Figshare: Underlying data for ‘Problem-based learning in-class of research methods: Development, application and evaluation’. https://doi.org/10.6084/m9.figshare.17087747 ^40^

Data are available under the terms of the Creative Commons Attribution 4.0 International license (CC-BY 4.0)

## Consent

Written informed consent for publication of the participants’ details was obtained from the participants.

## Author contributions

YR – Investigation, Validation, Data Curation, Visualization, and Writing – Original Draft Preparation

NKS – Supervision, Conceptualization, Methodology, Project Administration – Review & Editing

YK – Supervision, Conceptualization, Project Administration and Writing – Review & Editing

DH – Data Collections, Writing –Review & Editing
