# Supplementary material for: Problem-based learning in research method courses: development, application and evaluation
Source: F1000Res. 2023 Dec 11;11:378. Originally published 2022 Mar 31. [Version 2] doi: 10.12688/f1000research.75985.2 (PMC10804049; doi:10.12688/f1000research.75985.2)
Supplement: Peer review history

# Reviewer report: Reviewer response for version 2

**DOI:** 10.5256/f1000research.159886.r229124

**Published:** 2024-01-22

**Authors:** Achmad Samsudin

**Affiliations:** 1 Department of Physics Education, Universitas Pendidikan Indonesia, Bandung, Indonesia

The method in the abstract section consists of research methods, samples and their characteristics, instruments and data analysis techniques.

Is the work clearly and accurately presented and does it cite the current literature?

Yes

If applicable, is the statistical analysis and its interpretation appropriate?

Yes

Are all the source data underlying the results available to ensure full reproducibility?

Yes

Is the study design appropriate and is the work technically sound?

Partly

Are the conclusions drawn adequately supported by the results?

Yes

Are sufficient details of methods and analysis provided to allow replication by others?

Partly

Reviewer Expertise:

Education

I confirm that I have read this submission and believe that I have an appropriate level of expertise to confirm that it is of an acceptable scientific standard.

# Reviewer report: Reviewer response for version 2

**DOI:** 10.5256/f1000research.159886.r229123

**Published:** 2023-12-18

**Authors:** Dean Iliev

**Affiliations:** 1 University St. Kliment Ohridski, Bitola, North Macedonia

I am satisfied with the undertaken steps by the authors, so I wish them all the best.

Is the work clearly and accurately presented and does it cite the current literature?

Yes

If applicable, is the statistical analysis and its interpretation appropriate?

Yes

Are all the source data underlying the results available to ensure full reproducibility?

Yes

Is the study design appropriate and is the work technically sound?

Yes

Are the conclusions drawn adequately supported by the results?

Yes

Are sufficient details of methods and analysis provided to allow replication by others?

Yes

Reviewer Expertise:

Methodology of Research, Didactics, Methodology of action research, Life-long learning of teachers, Social pedagogy

I confirm that I have read this submission and believe that I have an appropriate level of expertise to confirm that it is of an acceptable scientific standard.

# Reviewer report: Reviewer response for version 1

**DOI:** 10.5256/f1000research.79933.r145910

**Published:** 2023-11-21

**Authors:** Achmad Samsudin

**Affiliations:** 1 Department of Physics Education, Universitas Pendidikan Indonesia, Bandung, Indonesia

**Abstract:**

- Methods: The research method is not yet clear. This section should include the method, is the research qualitative, quantitative, survey, case study, or what?
- Result: How much is it worth? Include the resulting value for the category earned.

**Introduction: **

- Adding research schemes, or research concept maps may be useful.

**Methods:**

- Design: The research method is not yet clear. This section should include the method, is the research qualitative, quantitative, survey, case study, or what?
- Participants: It's best to present it in tabular form for (a total of 40 students including nine males and thirty-one females, thirteen of whom had high abilities, eighteen were moderately capable and nine had low abilities. Information on the grouping based on gender and students’ ability levels was obtained from the Cumulative Achievement Index/GPA) and for gain categories.
- Add data analysis from IBM SPSS to the abstract.

**Conclusions:**

- Add value for each result you get.

Is the work clearly and accurately presented and does it cite the current literature?

Yes

If applicable, is the statistical analysis and its interpretation appropriate?

Yes

Are all the source data underlying the results available to ensure full reproducibility?

Yes

Is the study design appropriate and is the work technically sound?

Partly

Are the conclusions drawn adequately supported by the results?

Yes

Are sufficient details of methods and analysis provided to allow replication by others?

Partly

Reviewer Expertise:

Education

I confirm that I have read this submission and believe that I have an appropriate level of expertise to confirm that it is of an acceptable scientific standard, however I have significant reservations, as outlined above.

# Response

**Published:** 2023-12-05

**Authors:** Yani Ramdani

**Affiliations:** Universitas Islam Bandung, Indonesia

I am Author

**Abstract:**

- Methods: The research method is not yet clear. This section should include the method, is the research qualitative, quantitative, survey, case study, or what?

This research was a case study to report the integration of scientific report writing in research methods courses through PBL at the Bandung Islamic University, Indonesia. PBL is carried out by involving students in research-related tasks with the following stages: writing research questions, determining research design, collecting data, analyzing data, and presenting research results.

- Result: How much is it worth? Include the resulting value for the category earned.

The research results showed that there was an increase in scientific writing skills in the good category. Normalized gain for indicators of increasing accuracy using research report formats, suitability for using literature and research methods is 0.064 and 0.209, including the low category, and the plagiarism level is 0.509, including the medium category.

**Introduction: **

- Adding research schemes, or research concept maps may be useful.

This article examines the results of basic research to apply the PBL method in research methods courses in the mathematics study program at the Islamic University of Bandung Indonesia. In general, in the Undergraduate Program (S1) curriculum in Indonesia, every student is required to write a scientific work called a thesis to complete their study period. To equip students to write a thesis, students must take research methods courses. The general aim of this course is to teach students how to present research results both orally and in writing using the style and form of each university. PBL allows students to construct new knowledge by connecting previous knowledge more easily. 16 PBL allows students to search for information, solve problems, make decisions, work in groups, write reports, make presentations, be independent and responsible in facing complex problems from the start. real life.17

**Methods:**

- Design: The research method is not yet clear. This section should include the method, is the research qualitative, quantitative, survey, case study, or what?

This research is a case study. The participants were third-year students at an Universitas Islam Bandung Indonesia (a total of 40 students including nine males and thirty-one females, thirteen of whom had high abilities, eighteen were moderately capable and nine had low abilities. Information on the grouping based on gender and students' ability levels was obtained from the Cumulative Achievement Index/GPA) enrolled in the courses of research methods. In general, each student had different abilities in thinking and communicating. One of the differences could be seen from the value of the GPA. GPA was the measure performance of student at the academic field which was obtained by combining all the grades of the courses that had been taken up to a certain semester. ^34^  All students met the requirements agreed to: (1) case presentation, (2) conduct discussions, (3) make a summary, (4) participate in online learning, and (5) compile scientific reports.

- Participants: It's best to present it in tabular form for (a total of 40 students including nine males and thirty-one females, thirteen of whom had high abilities, eighteen were moderately capable and nine had low abilities. Information on the grouping based on gender and students’ ability levels was obtained from the Cumulative Achievement Index/GPA) and for gain categories.

Table 1. Data Description based Gender

 Gender

 Frequency

 Relative Frequency (%)

 Male

 9

 22,5

 Female

 31

 77,5

 Total

 40

 100

 Tabel 2. Data Description based Ability

 Ability

 Frequency

 Relative Frequency (%)

 High

 13

 32,5

 Middle

 18

 45,0

 Low

 9

 22,5

 Total

 40

 100

- Add data analysis from IBM SPSS to the abstract.

The research results showed that there was an increase in scientific writing skills in the good category. Normalized gain for indicators of increasing accuracy using research report formats, suitability for using literature and research methods is 0.064 and 0.209, including the low category, and the plagiarism level is 0.509, including the medium category.

**Conclusions:**

- Add value for each result you get.

The results of this research indicate that integrating research report writing through the PBL approach in research methods classes can develop students' abilities in compiling research reports with a significance value of 0.00. This integration is one of the best ways to engage and apply research methods theory directly in student research. The improvement in overall learning outcomes at the end of the positive PBL process indicates that integrated teaching is an effective way to reduce student barriers in compiling research reports. Students also realize that writing scientific reports is a learning process whose mastery of skills must be done through experience. Finally, some students feel trained and motivated in designing, conducting experiments, processing, and analyzing data. Based on the findings of this study, the PBL approach is recommended to support research at the undergraduate level. To ensure the quality of scientific reports, the support for scientific writing needs to get better attention. Supervision can involve other lecturers to form peer teaching. Peer support is developed to form a mutually supportive learning community.

- **Is the work clearly and accurately presented and does it cite the current literature?**

Yes

- **Is the study design appropriate and is the work technically sound?**

Partly

- **Are sufficient details of methods and analysis provided to allow replication by others?**

Partly

- **If applicable, is the statistical analysis and its interpretation appropriate?**

Yes

- **Are all the source data underlying the results available to ensure full reproducibility?**

Yes

- **Are the conclusions drawn adequately supported by the results?**

Yes

 Competing Interests

*No competing interests were disclosed.*

# Reviewer report: Reviewer response for version 1

**DOI:** 10.5256/f1000research.79933.r190225

**Published:** 2023-09-18

**Authors:** Dean Iliev

**Affiliations:** 1 University St. Kliment Ohridski, Bitola, North Macedonia

The topic of the article was chosen based on the experience of people who know this issue. The problem of studying research methodology exists everywhere in higher education and in the education of future teachers. Anyone who wants to improve their work and is concerned with developing the research competencies of their students will benefit from this research. The research is simple and limited in terms of research sample, but offers a sufficient basis for replication in different settings, for the same purpose. If the methodology in the paper were more complex and the statistics at a higher level, it would reduce its use and application by a greater number of our colleagues who are looking for a way to improve the study of research methodology in their classroom. I encourage other researchers and practitioners to follow this example and to create and conduct research on the impact of other contemporary learning and teaching strategies in advancing research methodology learning processes.

Is the work clearly and accurately presented and does it cite the current literature?

Yes

If applicable, is the statistical analysis and its interpretation appropriate?

Yes

Are all the source data underlying the results available to ensure full reproducibility?

Yes

Is the study design appropriate and is the work technically sound?

Yes

Are the conclusions drawn adequately supported by the results?

Yes

Are sufficient details of methods and analysis provided to allow replication by others?

Yes

Reviewer Expertise:

Methodology of Research, Didactics, Methodology of action research, Life-long learning of teachers, Social pedagogy

I confirm that I have read this submission and believe that I have an appropriate level of expertise to confirm that it is of an acceptable scientific standard.
